# The inverses of tails of the Riemann zeta function

**DOI:** 10.1186/s13660-018-1743-6

**Published:** 2018-07-03

**Authors:** Donggyun Kim, Kyunghwan Song

**Affiliations:** Department of Mathematics, Korea University, Seoul, Republic of Korea

**Keywords:** Riemann zeta function, Tails of Riemann zeta function, Inverses of tails of the Riemann zeta function, 11M06, 11B83

## Abstract

We present some bounds of the inverses of tails of the Riemann zeta function on $0 < s < 1$ and compute the integer parts of the inverses of tails of the Riemann zeta function for $s=\frac{1}{2}, \frac{1}{3}$, and $\frac{1}{4}$.

## Introduction

The Riemann zeta function $\zeta (s)$ in the real variable *s* was introduced by Euler [2] in connection with questions about the distribution of prime numbers. Later Riemann [6] derived deeper results about a dual correspondence between the distribution of prime numbers and the complex zeros of $\zeta (s)$ in the complex variable *s*. In these developments, he asserted that all the non-trivial zeros of $\zeta (s)$ are on the line $\operatorname{Re}(s) = \frac{1}{2}$, and this has been one of the most important unsolved problems in mathematics, called the Riemann hypothesis. A vast amount of research on calculation of $\zeta (s)$ on the line $\operatorname{Re}(s) = \frac{1}{2}$, which is called the critical line, and on the strip $0 < \operatorname{Re}(s) < 1$, which is called the critical strip, has been conducted using various methods [1].

The *Riemann zeta function* and a *tail of the Riemann zeta function from* *n* for an integer $n \geq 1$ are defined, respectively, by: for $\operatorname{Re}(s) > 1$,

$$ \zeta (s) = \sum_{k=1}^{\infty } \frac{1}{k^{s}} \quad \mbox{and} \quad \zeta_{n} (s) = \sum _{k=n}^{\infty } \frac{1}{k^{s}}, $$

and for $0<\operatorname{Re}(s)<1$,

$$ \zeta (s) =\frac{1}{1-2^{1-s}} \sum_{k=1}^{\infty } \frac{(-1)^{k+1}}{k ^{s}} \quad \mbox{and} \quad \zeta_{n} (s) = \frac{1}{1-2^{1-s}} \sum_{k=n}^{\infty } \frac{(-1)^{k+1}}{k^{s}}. $$

To understand the values of $\zeta (s)$, it would be helpful to understand the values of tails of $\zeta (s)$, for example, the integer parts of their inverses $[ \zeta_{n} (s)^{-1} ] $, where $[x]$ denotes the greatest integer that is less than or equal to *x*.

Some values of $[ \zeta_{n} (s)^{-1} ] $ for small positive integers *s* have become known recently. Xin [7] showed that for $s = 2$ and 3,

$$ \bigl[ \zeta_{n} (2)^{-1} \bigr] =n-1 \quad \mbox{and} \quad \bigl[ \zeta_{n} (3)^{-1} \bigr] =2n(n-1). $$

For $s=4$, Xin and Xiaoxue [8] showed that

$$ \bigl[ \zeta_{n} (4)^{-1} \bigr] = 3n^{3} -5n^{2} + 4n-1+ \biggl[ \frac{(2n+1)(n-1)}{4} \biggr] $$

for any integer $n \geq 2$, and Xu [9] showed that for $s = 5$,

$$ \bigl[ \zeta_{n} (5)^{-1} \bigr] = 4n^{4} - 8n^{3} + 9n^{2} - 5n + \biggl[ \frac{(n+1)(n-2)}{3} \biggr] $$

for any integer $n \geq 4$. Hwang and Song [3] provided an alternative proof of the case when $s = 5$ and a formula when $s = 6$ as follows. For an integer *n*, write $n_{48}$ for the remainder when *n* is divided by 48, then

$$\begin{aligned} & \bigl[ \zeta_{n} (6)^{-1} \bigr] \\ &\quad = \textstyle\begin{cases} 5n^{5} - \frac{25}{2} n^{4} +\frac{75}{4} n^{3} -\frac{125}{8} n^{2} + \frac{185}{48}n - \frac{5 n_{48}}{48} - [ \frac{35-5 n_{48}}{48} ] ,&\mbox{if~$n$~is even}, \\ 5n^{5} - \frac{25}{2} n^{4} +\frac{75}{4} n^{3} -\frac{125}{8} n^{2} +\frac{185}{48}n - \frac{5 n_{48}+18}{48} - [ \frac{17-5 n_{48}}{48} ] ,& \mbox{if~$n$~is odd} \end{cases}\displaystyle \end{aligned}$$

for any integer $n \geq 829$. For the integer *s* greater than 6, no such a formula is known.

There are other interesting results related to this theme such as bounds of $\zeta (3)$ in greater precision in [4] and [5].

We study the inverses of tails of the Riemann zeta function $\zeta_{n}(s)^{-1}$ for *s* on the critical strip $0 < s <1$. The following notation is needed to explain our results.

### Definition 1

For any positive integer *n* and real number *s* with $0< s<1$, we define

$$\begin{aligned} A_{n,s}= \biggl( \frac{1}{n^{s}} - \frac{1}{(n+1)^{s}} \biggr) + \biggl( \frac{1}{(n+2)^{s}} - \frac{1}{(n+3)^{s}} \biggr) + \cdots \end{aligned}$$

and

$$\begin{aligned} B_{n,s} = \biggl( -\frac{1}{n^{s}} + \frac{1}{(n+1)^{s}} \biggr) + \biggl( -\frac{1}{(n+2)^{s}} + \frac{1}{(n+3)^{s}} \biggr) + \cdots . \end{aligned}$$

Now the tail of the Riemann zeta function for $0< s<1$ can be written as follows:

1 $$ \zeta_{n}(s) = \textstyle\begin{cases} - \frac{1}{1-2^{1-s}} A_{n,s}, &\mbox{if~$n$~is even}, \\ - \frac{1}{1-2^{1-s}} B_{n,s},& \mbox{if~$n$~is odd}. \end{cases} $$

In this paper, we present the bounds of $A_{n,s}^{-1}$ and $B_{n,s} ^{-1}$, hence the bounds of the inverses of tails of the Riemann zeta function $\zeta_{n}(s)^{-1}$ for $0< s<1$ in Sect. 2.1, and compute the values $[ A_{n,s}^{-1} ] $ and $[ B_{n,s}^{-1} ] $, hence the values of the inverses of tails of the Riemann zeta function $[ \frac{1}{1-2^{1-s}}\zeta_{n}(s) ^{-1} ] $ for $s= \frac{1}{2},\, \frac{1}{3}$, and $\frac{1}{4}$ in Sect. 2.2.

## Main results

### The bounds of the inverses of $\zeta_{n}(s)$ for $0< s<1$

In this section, we present the bounds of $A_{n,s}^{-1}$ and $B_{n,s}^{-1}$ in Definition 1, hence the bounds of the inverses of tails of the Riemann zeta function $\zeta_{n}(s)^{-1}$ for $0< s<1$.

#### Proposition 1

*Let* *s* *be a real number with* $0< s<1$. *Then*, *for any positive even number* *n*,

$$\begin{aligned}& 2(n-1)^{s} < A^{-1}_{n,s} < 2{n}^{s}, \end{aligned}$$

*and for any positive odd number* *n*,

$$\begin{aligned}& -2{n}^{s} < B^{-1}_{n,s} < -2(n-1)^{s}. \end{aligned}$$

#### Proof

Let *n* be a positive even number. For every positive integer *k*, it is easy to see that

$$\begin{aligned} &\biggl( \frac{1}{(n+1+2k)^{s}} - \frac{1}{(n+2+2k)^{s}} \biggr) \\ &\quad < \biggl( \frac{1}{(n+2k)^{s}} - \frac{1}{(n+1+2k)^{s}} \biggr) \\ &\quad < \biggl( \frac{1}{(n-1+2k)^{s}} - \frac{1}{(n+2k)^{s}} \biggr) . \end{aligned}$$

The summations of each term over *k* give

$$\begin{aligned}& A_{n+1,s} < A_{n,s} < A_{n-1,s} \end{aligned}$$

and

$$\begin{aligned}& \frac{1}{2} (A_{n+1,s} + A_{n,s}) < A_{n,s} < \frac{1}{2} (A_{n-1,s} + A_{n,s}). \end{aligned}$$

Therefore, we have

$$\begin{aligned}& \frac{1}{2n^{s}} < A_{n,s} < \frac{1}{2(n-1)^{s}}, \end{aligned}$$

which gives the first statement.

The second statement can be shown similarly. □

Since every proof of the case when *n* is an odd number is analogous to that of the case when *n* is an even number, we omit all the proofs of the odd number cases in this paper.

Now we find tighter bounds for $A^{-1}_{n,s}$ and $B^{-1}_{n,s}$.

#### Proposition 2

*Let* *s* *be a real number with* $0< s<1$. *Then*, *for any positive even number* *n*,

$$ 2 \biggl( n-\frac{1}{2} \biggr) ^{s} < A^{-1}_{n,s}, $$

*and for any positive odd number* *n*,

$$ B^{-1}_{n,s} < -2 \biggl( n-\frac{1}{2} \biggr) ^{s} . $$

#### Proof

Let *n* be a positive even number. We will show that

$$\begin{aligned}& A_{n,s} < \frac{1}{2 ( n-\frac{1}{2} ) ^{s}}. \end{aligned}$$

Rewriting each of the both sides as a series

$$\begin{aligned} A_{n,s} & = \sum_{k=\frac{n}{2}}^{\infty } \biggl( \frac{1}{(2k)^{s}} - \frac{1}{(2k+1)^{s}} \biggr) \end{aligned}$$

and

$$\begin{aligned} \frac{1}{2(n-\frac{1}{2})^{s}} & = \sum_{k=\frac{n}{2}}^{\infty } \biggl( \frac{1}{2(2k-\frac{1}{2})^{s}} - \frac{1}{2( 2k + \frac{3}{2})^{s}} \biggr) , \end{aligned}$$

we will show that for any positive integer *k*,

$$\begin{aligned}& \frac{1}{(2k)^{s}} - \frac{1}{(2k+1)^{s}} < \frac{1}{2 ( 2k - \frac{1}{2} ) ^{s}} - \frac{1}{2 ( 2k+\frac{3}{2} ) ^{s}}. \end{aligned}$$

For this, we let

$$ f(x) = \biggl( \frac{1}{2(2x-\frac{1}{2})^{s}} - \frac{1}{2(2x+ \frac{3}{2})^{s}} \biggr) - \biggl( \frac{1}{(2x)^{s}} - \frac{1}{(2x+1)^{s}} \biggr) $$

and will show that $f(x)$ is positive for $x \ge 1$ and $0< s<1$. With

$$ g(x) = \frac{1}{2(2x-\frac{1}{2})^{s}} + \frac{1}{2(2x+\frac{1}{2})^{s}} - \frac{1}{(2x)^{s}}, $$

we have $f(x) = g(x) -g(x+\frac{1}{2})$. Consider the derivative of $g(x)$:

$$ g'(x) =-2s \biggl( \frac{1}{2(2x-\frac{1}{2})^{s+1}} + \frac{1}{2(2x+ \frac{1}{2})^{s+1}} - \frac{1}{(2x)^{s+1}} \biggr) . $$

Since the function $\frac{1}{x^{s+1}}$ is convex, we obtain that

$$ \frac{1}{2(2x-\frac{1}{2})^{s+1}} + \frac{1}{2(2x+\frac{1}{2})^{s+1}} - \frac{1}{(2x)^{s+1}} \geq 0, $$

and therefore $g'(x)$ is negative, that is, $g(x)$ is decreasing. We conclude that $f(x)$ is positive, which gives the statement. □

#### Proposition 3

*Let* *s* *be a real number with* $0< s<1$. *Then*, *for any positive even number* *n*,

$$ A^{-1}_{n,s} < 2 \biggl( n-\frac{1}{4} \biggr) ^{s}, $$

*and for any positive odd number* *n*,

$$ -2 \biggl( n-\frac{1}{4} \biggr) ^{s}< B^{-1}_{n,s}. $$

#### Proof

Let *n* be a positive even number. We will show that

$$\begin{aligned}& \frac{1}{2 ( n-\frac{1}{4} ) ^{s}} < A_{n,s}. \end{aligned}$$

Rewriting each of the both sides as a series

$$\begin{aligned} A_{n,s} & = \sum_{k=\frac{n}{2}}^{\infty } \biggl( \frac{1}{(2k)^{s}} - \frac{1}{(2k+1)^{s}} \biggr) \end{aligned}$$

and

$$\begin{aligned} \frac{1}{2(n-\frac{1}{4})^{s}} & = \sum_{k=\frac{n}{2}}^{\infty } \biggl( \frac{1}{2(2k-\frac{1}{4})^{s}} - \frac{1}{2(2k+\frac{7}{4})^{s}} \biggr) , \end{aligned}$$

we need to show that for any positive integer *k*,

$$\begin{aligned}& \frac{1}{2(2k-\frac{1}{4})^{s}} - \frac{1}{2(2k+\frac{7}{4})^{s}} < \frac{1}{(2k)^{s}} - \frac{1}{(2k+1)^{s}}. \end{aligned}$$

For this, we let

$$ f(x) = \biggl( \frac{1}{(2x)^{s}} - \frac{1}{(2x+1)^{s}} \biggr) - \biggl( \frac{1}{2(2x - \frac{1}{4})^{s}} - \frac{1}{2(2x+\frac{7}{4})^{s}} \biggr) . $$

We check that $f(1)>0$ and now we will show that $f(x)$ is positive for $x \geq 2$ and $0 < s < 1$. With

$$ g(x) = \frac{1}{(2x)^{s}} - \biggl( \frac{1}{2(2x-\frac{1}{4})^{s}} + \frac{1}{2(2x+ \frac{3}{4})^{s}} \biggr) , $$

we have $f(x) = g(x) - g(x+\frac{1}{2})$, so we only need to show that $g(x)$ is decreasing. Consider the derivative of $g(x)$:

$$\begin{aligned} g'(x) & = s \biggl( - \frac{2}{(2x)^{s+1}} + \biggl( \frac{1}{ ( 2x- \frac{1}{4} ) ^{s+1}} + \frac{1}{ ( 2x+\frac{3}{4} ) ^{s+1}} \biggr) \biggr) \\ & = s \biggl( \biggl( \frac{1}{ ( 2x-\frac{1}{4} ) ^{s+1}} - \frac{1}{(2x)^{s+1}} \biggr) - \biggl( \frac{1}{(2x)^{s+1}} - \frac{1}{ ( 2x+\frac{3}{4} ) ^{s+1}} \biggr) \biggr) . \end{aligned}$$

Since the function $\frac{1}{x^{s+1}}$ is decreasing and convex, by comparing slopes at $(2x-\frac{1}{4})$ and $(2x+\frac{3}{4})$, we obtain

$$\begin{aligned}& \frac{1}{ ( 2x-\frac{1}{4} ) ^{s+1}} - \frac{1}{(2x)^{s+1}} < \frac{1}{4} (s+1) \frac{1}{ ( 2x-\frac{1}{4} ) ^{s+2}} \end{aligned}$$

and

$$\begin{aligned}& \frac{1}{(2x)^{s+1}} - \frac{1}{ ( 2x+\frac{3}{4} ) ^{s+1}} > \frac{1}{4} (s+1) \frac{3}{ ( 2x+\frac{3}{4} ) ^{s+2}}. \end{aligned}$$

Therefore,

$$\begin{aligned}& g'(x) < \frac{1}{4} s (s+1) \biggl( \frac{1}{ ( 2x-\frac{1}{4} ) ^{s+2}} - \frac{3}{ ( 2x+\frac{3}{4} ) ^{s+2}} \biggr) . \end{aligned}$$

Consider $h(x,s):= \frac{1}{3} ( \frac{2x+3/4}{2x-1/4} ) ^{s+2}$, which is the ratio of two terms on the right-hand side of the above expression. We check that $h(x,s) < 1$ for $x \geq 2$ and $0< s<1$. Since $h(2,1) = 6859/10\text{,}125$ and $\lim_{x \rightarrow \infty } h(x,s) = \frac{1}{3}$ for $0< s<1$, we obtain that $g'(x)$ is negative and, therefore, $g(x)$ is decreasing, which gives the statement. □

We combine the results of Proposition 2 and Proposition 3.

#### Theorem 1

*Let* *s* *be a real number with* $0< s<1$. *Then*, *for any positive even number* *n*,

$$\begin{aligned}& 2 \biggl( n-\frac{1}{2} \biggr) ^{s} < A^{-1}_{n,s} < 2 \biggl( n - \frac{1}{4} \biggr) ^{s}, \end{aligned}$$

*and for any positive odd number* *n*,

$$\begin{aligned}& -2 \biggl( n-\frac{1}{4} \biggr) ^{s} < B^{-1}_{n,s} < -2 \biggl( n - \frac{1}{2} \biggr) ^{s}. \end{aligned}$$

We express these bounds in terms of $\zeta_{n}(s)$ using expression (1).

#### Corollary 1

*Let* *s* *be a real number with* $0< s<1$. *Then*, *for any positive even number* *n*,

$$\begin{aligned}& 2\bigl(1-2^{1-s}\bigr) \biggl( n-\frac{1}{4} \biggr) ^{s} < \zeta_{n}(s)^{-1} < 2\bigl(1-2^{1-s} \bigr) \biggl( n - \frac{1}{2} \biggr) ^{s}, \end{aligned}$$

*and for any positive odd number* *n*,

$$\begin{aligned}& -2\bigl(1-2^{1-s}\bigr) \biggl( n-\frac{1}{2} \biggr) ^{s} < \zeta_{n}(s)^{-1} < -2\bigl(1-2^{1-s} \bigr) \biggl( n - \frac{1}{4} \biggr) ^{s}. \end{aligned}$$

Furthermore, we have tighter bounds of $A^{-1}_{n,s}$ and $B^{-1}_{n,s}$ for a sufficiently large number *n*.

#### Theorem 2

*For any positive number* *ϵ* *and any real number* *s* *with* $0< s<1$,

$$\begin{aligned}& 2 \biggl( n-\frac{1}{2} \biggr) ^{s} < A^{-1}_{n,s} < 2 \biggl( n - \frac{1}{2} + \epsilon \biggr) ^{s} \end{aligned}$$

*for a sufficiently large even number* *n* *and*

$$\begin{aligned}& -2 \biggl( n-\frac{1}{2} + \epsilon \biggr) ^{s} < B^{-1}_{n,s} < -2 \biggl( n - \frac{1}{2} \biggr) ^{s} \end{aligned}$$

*for a sufficiently large odd number n*.

#### Proof

From Theorem 1, it suffices to show that for a sufficiently large even number *n*,

$$\begin{aligned}& \frac{1}{2 ( n-\frac{1}{2} + \epsilon ) ^{s}} < A_{n,s}. \end{aligned}$$

Rewriting each of the both sides as a series

$$\begin{aligned} A_{n,s} & = \sum_{k=\frac{n}{2}}^{\infty } \biggl( \frac{1}{(2k)^{s}} - \frac{1}{(2k+1)^{s}} \biggr) \end{aligned}$$

and

$$\begin{aligned} \frac{1}{2 ( n - \frac{1}{2} + \epsilon ) ^{s}} & = \sum_{k=\frac{n}{2}}^{\infty } \biggl( \frac{1}{2 ( 2k-\frac{1}{2} + \epsilon ) ^{s}} - \frac{1}{2 ( 2k+\frac{3}{2} + \epsilon ) ^{s}} \biggr) , \end{aligned}$$

we need to show that for a sufficiently large even number *n* and every integer $k \geq \frac{n}{2}$,

$$\begin{aligned}& \frac{1}{2 ( 2k - \frac{1}{2} + \epsilon ) ^{s}} - \frac{1}{2 ( 2k + \frac{3}{2} + \epsilon ) ^{s}} < \frac{1}{(2k)^{s}} - \frac{1}{(2k+1)^{s}}. \end{aligned}$$

For this, let

$$ f(x) = \biggl( \frac{1}{(2x)^{s}} - \frac{1}{(2x+1)^{s}} \biggr) - \biggl( \frac{1}{2(2x- \frac{1}{2} + \epsilon )^{s}} - \frac{1}{2(2x+\frac{3}{2} + \epsilon )^{s}} \biggr) , $$

and we will show that $f(x)$ is positive for $x \geq x_{0}$, where $x_{0}$ is a sufficiently large number. With

$$ g(x)= \frac{1}{(2x)^{s}} - \biggl( \frac{1}{2(2x-\frac{1}{2} + \epsilon )^{s}} + \frac{1}{2(2x+\frac{1}{2} + \epsilon )^{s}} \biggr) , $$

we have that $f(x) = g(x) - g(x+\frac{1}{2})$, so we only need to show that $g(x)$ is decreasing. Consider the derivative of $g(x)$:

$$\begin{aligned} g'(x) & = s \biggl( -\frac{2}{(2x)^{s+1}} + \frac{1}{ ( 2x - \frac{1}{2} + \epsilon ) ^{s+1}} + \frac{1}{ ( 2x + \frac{1}{2} + \epsilon ) ^{s+1}} \biggr) \\ & = s \biggl( \biggl( \frac{1}{ ( 2x - \frac{1}{2} + \epsilon ) ^{s+1}} - \frac{1}{(2x)^{s+1}} \biggr) - \biggl( \frac{1}{(2x)^{s+1}} - \frac{1}{ ( 2x + \frac{1}{2} + \epsilon ) ^{s+1}} \biggr) \biggr) . \end{aligned}$$

Since $\frac{1}{x^{s+1}}$ is decreasing and convex, by comparing slopes at $(2x - \frac{1}{2} + \epsilon )$ and $(2x + \frac{1}{2} + \epsilon )$, we obtain

$$\begin{aligned}& \frac{1}{ ( 2x - \frac{1}{2} + \epsilon ) ^{s+1}} - \frac{1}{(2x)^{s+1}} < (s+1) \frac{\frac{1}{2} - \epsilon }{ ( 2x - \frac{1}{2} + \epsilon ) ^{s+2}} \end{aligned}$$

and

$$\begin{aligned}& \frac{1}{(2x)^{s+1}} - \frac{1}{ ( 2x+\frac{1}{2} + \epsilon ) ^{s+1}} > (s+1) \frac{\frac{1}{2} + \epsilon }{ ( 2x+\frac{1}{2} + \epsilon ) ^{s+2}}. \end{aligned}$$

Therefore

$$\begin{aligned}& g'(x) < s(s+1) \biggl( \frac{\frac{1}{2} - \epsilon }{ ( 2x - \frac{1}{2} + \epsilon ) ^{s+2}} - \frac{\frac{1}{2} + \epsilon }{ ( 2x + \frac{1}{2} + \epsilon ) ^{s+2}} \biggr) . \end{aligned}$$

Consider $h(x):= \frac{\frac{1}{2} - \epsilon }{\frac{1}{2} + \epsilon } ( \frac{2x+\frac{1}{2} + \epsilon }{2x - \frac{1}{2} + \epsilon } ) ^{s+2}$, which is the ratio of two terms on the right-hand side of the above expression. We need to show that $h(x) < 1$ for every $x > x_{0}$, where $x_{0}$ is a sufficiently large number. We check that

$$\begin{aligned}& h(x) < 1 \quad \iff \quad \frac{2x+\frac{1}{2} + \epsilon }{2x - \frac{1}{2} + \epsilon } < \biggl( \frac{\frac{1}{2} + \epsilon }{\frac{1}{2} - \epsilon } \biggr) ^{\frac{1}{s+2}}. \end{aligned}$$

For any $\epsilon > 0$ and $0 < s < 1$, we have that $1 < ( \frac{ \frac{1}{2} + \epsilon }{\frac{1}{2} - \epsilon } ) ^{1/(s+2)}$ and $\frac{2x + \frac{1}{2} + \epsilon }{2x - \frac{1}{2} + \epsilon }$ is larger than 1, decreasing and converges to 1 as *x* goes to infinity, so there is $x_{0}$ such that, for every $x > x_{0}$, $h(x) < 1$. Therefore the proof is complete. □

We express these bounds in terms of $\zeta_{n}(s)$ using expression (1).

#### Corollary 2

*For any positive number* *ϵ* *and any real number* *s* *with* $0< s<1$, *we have*

$$\begin{aligned}& 2\bigl(1-2^{1-s}\bigr) \biggl( n-\frac{1}{2} + \epsilon \biggr) ^{s} < \zeta_{n}(s)^{-1} < 2\bigl(1-2^{1-s} \bigr) \biggl( n-\frac{1}{2} \biggr) ^{s}, \end{aligned}$$

*for a sufficiently large even number* *n* *and*

$$\begin{aligned}& -2\bigl(1-2^{1-s}\bigr) \biggl( n-\frac{1}{2} \biggr) ^{s} < \zeta_{n}(s)^{-1} < -2\bigl(1-2^{1-s} \bigr) \biggl( n - \frac{1}{2} + \epsilon \biggr) ^{s} \end{aligned}$$

*for a sufficiently large odd number* *n*.

### The value of the inverse of $\zeta_{n}(s)$ for $s=\frac{1}{2}, \frac{1}{3}$, and $\frac{1}{4}$

We study firstly the value of the inverse of $\zeta_{n}(\frac{1}{2})$, where $\zeta_{n}(\frac{1}{2})$ is the tail of the Riemann zeta function from *n* at $s=\frac{1}{2}$.

#### Theorem 3

*For any positive even number* *n*,

$$\begin{aligned}& \bigl[A^{-1}_{n, 1/2}\bigr] = \biggl[ 2 \biggl( n- \frac{1}{2} \biggr) ^{1/2} \biggr] , \end{aligned}$$

*and for any positive odd number* *n*,

$$\begin{aligned}& \bigl[B^{-1}_{n, 1/2}\bigr] = \biggl[ -2 \biggl( n- \frac{1}{2} \biggr) ^{1/2} \biggr] . \end{aligned}$$

#### Proof

Let *n* be a positive even number. By Theorem 1, we have that

$$\begin{aligned}& 2 \biggl( n - \frac{1}{2} \biggr) ^{1/2} < A^{-1}_{n,1/2} < 2 \biggl( n - \frac{1}{4} \biggr) ^{1/2}. \end{aligned}$$

Note that $2(n - \frac{1}{4})^{1/2} - 2(n - \frac{1}{2})^{1/2} < 1$ for $n \geq 2$, and it implies that there is at most one integer in the open interval from $2(n-\frac{1}{2})^{1/2}$ to $2(n-\frac{1}{4})^{1/2}$. Suppose that there is an integer *h* in the open interval, i.e.,

$$ 2 \biggl( n - \frac{1}{2} \biggr) ^{1/2} < h < 2 \biggl( n - \frac{1}{4} \biggr) ^{1/2} \quad \mbox{or} \quad 4n-2 < h^{2} < 4n-1. $$

There is, however, no integer in the open interval from $4n-2$ to $4n-1$, therefore such an integer *h* does not exist. This gives the statement. □

We express this result in terms of $\zeta_{n}(s)$ using expression (1).

#### Corollary 3

*For any positive integer* *n*,

$$\begin{aligned}& \biggl[ \frac{1}{1-2^{1/2}} \zeta_{n} \biggl( \frac{1}{2} \biggr) ^{-1} \biggr] = \biggl[ (-1)^{n+1} 2 \biggl( n- \frac{1}{2} \biggr) ^{1/2} \biggr] . \end{aligned}$$

We study secondly the value of the inverse of $\zeta_{n}(\frac{1}{3})$, where $\zeta_{n}(\frac{1}{3})$ is the tail of the Riemann zeta function from *n* at $s=\frac{1}{3}$.

#### Theorem 4

*For any positive even number* *n*,

$$\begin{aligned}& \bigl[A^{-1}_{n, 1/3}\bigr] = \biggl[ 2 \biggl( n- \frac{1}{2} \biggr) ^{1/3} \biggr] , \end{aligned}$$

*and for any positive odd number* *n*,

$$\begin{aligned}& \bigl[B^{-1}_{n, 1/3}\bigr] = \biggl[ -2 \biggl( n- \frac{1}{2} \biggr) ^{1/3} \biggr] . \end{aligned}$$

#### Proof

Let *n* be a positive even number. By Theorem 1, we have that

$$\begin{aligned}& 2 \biggl( n - \frac{1}{2} \biggr) ^{1/3} < A^{-1}_{n,1/3} < 2 \biggl( n - \frac{1}{4} \biggr) ^{1/3}. \end{aligned}$$

Note that $2(n - \frac{1}{4})^{1/3} - 2(n - \frac{1}{2})^{1/3} < 1$ for $n \geq 2$, and it implies that there is at most one integer in the open interval from $2(n-\frac{1}{2})^{1/3}$ to $2(n-\frac{1}{4})^{1/3}$. Suppose that there is an integer *h* in the open interval, i.e.,

$$ 2\biggl(n - \frac{1}{2}\biggr)^{1/3} < h < 2\biggl(n - \frac{1}{4}\biggr)^{1/3} \quad \mbox{or} \quad 8n - 4 < h^{3} < 8n - 2. $$

This shows that the integer *h* is of the form $h = 2(n-\frac{3}{8})^{1/3}$. If we show $A^{-1}_{n,1/3} < 2(n-\frac{3}{8})^{1/3}$ or, equivalently, $\frac{1}{2(n-\frac{3}{8})^{1/3}} < A_{n,1/3}$, then our proof will be done. Let us rewrite

$$\begin{aligned} A_{n,1/3} & = \sum_{k=\frac{n}{2}}^{\infty } \biggl( \frac{1}{(2k)^{1/3}} - \frac{1}{(2k+1)^{1/3}} \biggr) \end{aligned}$$

and

$$\begin{aligned} \frac{1}{2(n-\frac{3}{8})^{1/3}} & = \sum_{k=\frac{n}{2}}^{\infty } \biggl( \frac{1}{2(2k-\frac{3}{8})^{1/3}} - \frac{1}{2(2k+\frac{13}{8})^{1/3}} \biggr) . \end{aligned}$$

Now it suffices to show that for any positive integer *k*,

$$\begin{aligned}& \frac{1}{2 ( 2k - \frac{3}{8} ) ^{1/3}} - \frac{1}{2 ( 2k + \frac{13}{8} ) ^{1/3}} < \frac{1}{(2k)^{1/3}} - \frac{1}{2 ( 2k +1 ) ^{1/3}}. \end{aligned}$$

For this, we let

$$ f(x) = \biggl( \frac{1}{(2x)^{1/3}} - \frac{1}{(2x+1)^{1/3}} \biggr) - \biggl( \frac{1}{2(2x - \frac{3}{8})^{1/3}} - \frac{1}{2(2x+ \frac{13}{8})^{1/3}} \biggr) , $$

and we will show that $f(x)$ is positive for any positive integer *x*.

We check that $f(1) = 0.00053\cdots$ and $f(2) = 0.00081\cdots$ , so it suffices to show $f(x) > 0$ for $x \geq 3$. With

$$ g(x) = \frac{1}{(2x)^{1/3}} - \biggl( \frac{1}{2(2x - \frac{3}{8})^{1/3}} + \frac{1}{2(2x + \frac{5}{8})^{1/3}}\biggr) , $$

we have that $f(x) = g(x) - g(x+\frac{1}{2})$, so we only need to show that $g(x)$ is decreasing for $x \ge 3$. Consider the derivative of $g(x)$:

$$\begin{aligned} g'(x) & = \frac{1}{3} \biggl( -\frac{2}{(2x)^{4/3}} + \frac{1}{ ( 2x - \frac{3}{8} ) ^{4/3}} + \frac{1}{ ( 2x + \frac{5}{8} ) ^{4/3}} \biggr) \\ & = \frac{1}{3} \biggl( \biggl( \frac{1}{ ( 2x -\frac{3}{8} ) ^{4/3}} - \frac{1}{(2x)^{4/3}} \biggr) - \biggl( \frac{1}{(2x)^{4/3}} - \frac{1}{ ( 2x + \frac{5}{8} ) ^{4/3}} \biggr) \biggr) . \end{aligned}$$

Since $\frac{1}{x^{4/3}}$ is decreasing and convex, by comparing slopes at $(2x - \frac{3}{8})$ and $(2x + \frac{5}{8})$, we obtain

$$\begin{aligned}& \frac{1}{ ( 2x - \frac{3}{8} ) ^{4/3}} - \frac{1}{(2x)^{4/3}} < 2\cdot \frac{3}{16} \cdot \frac{4}{3} \cdot \frac{1}{ ( 2x - \frac{3}{8} ) ^{7/3}} \end{aligned}$$

and

$$\begin{aligned}& \frac{1}{(2x)^{4/3}} - \frac{1}{ ( 2x + \frac{5}{8} ) ^{4/3}} > 2\cdot \frac{5}{16} \cdot \frac{4}{3} \cdot \frac{1}{ ( 2x + \frac{5}{8} ) ^{7/3}}. \end{aligned}$$

Therefore

$$\begin{aligned}& g'(x) < \frac{1}{18} \biggl( \frac{3}{ ( 2x - \frac{3}{8} ) ^{7/3}} - \frac{5}{ ( 2x + \frac{5}{8} ) ^{7/3}} \biggr) . \end{aligned}$$

Consider $h(x):= \frac{3}{5} ( \frac{2x + 5/8}{2x - 3/8} ) ^{7/3}$, which is the ratio of two terms of the right-hand side of the above expression. We check that $h(x) < 1$ for $x \geq 3$ because $h(3) = 0.87\cdots $ and $\lim_{x \rightarrow \infty } h(x) = \frac{3}{5}$ and $h'(x) < 0$ for $x \geq 3$. Hence we obtain that $g'(x)$ is negative and so $g(x)$ is decreasing for $x \geq 3$, which proves the statement. □

We express this result in terms of $\zeta_{n}(s)$ using expression (1).

#### Corollary 4

*For any positive integer* *n*,

$$\begin{aligned}& \biggl[ \frac{1}{1-2^{2/3}} \zeta_{n} \biggl( \frac{1}{3} \biggr) ^{-1} \biggr] = \biggl[ (-1)^{n+1} 2 \biggl( n- \frac{1}{2} \biggr) ^{1/3} \biggr] . \end{aligned}$$

We study lastly the value of the inverse of $\zeta_{n}(\frac{1}{4})$, which is the tail of the Riemann zeta function from *n* at $s= \frac{1}{4}$.

#### Theorem 5

*For any positive even number* *n*,

$$\begin{aligned}& \bigl[A^{-1}_{n, 1/4}\bigr] = \biggl[ 2 \biggl( n- \frac{1}{2} \biggr) ^{1/4} \biggr] , \end{aligned}$$

*and for any positive odd number* *n*,

$$\begin{aligned}& \bigl[B^{-1}_{n, 1/4}\bigr] = \biggl[ -2 \biggl( n- \frac{1}{2} \biggr) ^{1/4} \biggr] . \end{aligned}$$

#### Proof

Let *n* be a positive even number. By Theorem 1, we have that

$$\begin{aligned}& 2 \biggl( n - \frac{1}{2} \biggr) ^{1/4} < A^{-1}_{n,1/4} < 2 \biggl( n - \frac{1}{4} \biggr) ^{1/4}. \end{aligned}$$

Note that $2(n - \frac{1}{4})^{1/4} - 2(n - \frac{1}{2})^{1/4} < 1$ for $n \geq 2$, and it implies that there is at most one integer in the open interval from $2(n-\frac{1}{2})^{1/4}$ to $2(n-\frac{1}{4})^{1/4}$. Suppose that there is an integer *h* in the open interval, i.e.,

$$ 2\biggl(n-\frac{1}{2}\biggr)^{1/4} < h < 2\biggl(n - \frac{1}{4}\biggr)^{1/4}\quad \mbox{or}\quad 16n - 8 < h^{4} < 16n - 4. $$

This shows that the integer $h^{4}$ is one of the form $16n - 7$, $16n - 6$, or $16n - 5$. For any integer *h*, however, $h^{4} \equiv 0$ or $1 \pmod{16}$, hence such an integer *h* does not exist. Therefore this gives the statement. □

We express this result in terms of $\zeta_{n}(s)$ using expression (1).

#### Corollary 5

*For any positive integer* *n*,

$$\begin{aligned}& \biggl[ \frac{1}{1-2^{3/4}} \zeta_{n} \biggl( \frac{1}{4} \biggr) ^{-1} \biggr] = \biggl[ (-1)^{n+1} 2 \biggl( n- \frac{1}{2} \biggr) ^{1/4} \biggr] . \end{aligned}$$

We express the results of Theorems 3, 4, and 5 in a single statement.

#### Theorem 6

*For* $s = \frac{1}{2},\, \frac{1}{3}$, *or* $\frac{1}{4}$, *and for any positive even number* *n*,

$$\begin{aligned}& \bigl[A^{-1}_{n, s}\bigr] = \biggl[ 2 \biggl( n- \frac{1}{2} \biggr) ^{s} \biggr] , \end{aligned}$$

*and for any positive odd number* *n*,

$$\begin{aligned}& \bigl[B^{-1}_{n, s}\bigr] = \biggl[ -2 \biggl( n- \frac{1}{2} \biggr) ^{s} \biggr] . \end{aligned}$$

We express the results of Corollaries 3, 4, and 5 in a single statement.

#### Corollary 6

*For any positive integer* *n* *and* $s = \frac{1}{2},\, \frac{1}{3}$, *or* $\frac{1}{4}$,

$$\begin{aligned}& \biggl[ \frac{1}{1-2^{1-s}} \zeta_{n} ( s ) ^{-1} \biggr] = \biggl[ (-1)^{n+1} 2 \biggl( n-\frac{1}{2} \biggr) ^{s} \biggr] . \end{aligned}$$

## Conclusion

In this paper, we have presented the bounds of $A_{n,s}^{-1}$ and $B_{n,s}^{-1}$, hence the bounds of the inverses of tails of the Riemann zeta function $\zeta_{n}(s)^{-1}$ for $0< s<1$, and computed the values $[ A_{n,s}^{-1} ] $ and $[ B_{n,s}^{-1} ] $, hence the values of the inverses of tails of the Riemann zeta function $[ \frac{1}{1-2^{1-s}}\zeta_{n}(s) ^{-1} ] $ for $s= \frac{1}{2},\, \frac{1}{3}$, and $\frac{1}{4}$. For other values of *s*, for example $s=\frac{1}{5}$ or $\frac{2}{3} $, the values of $A_{n,s}$ and $B_{n,s}$ do not seem to have simple expressions.
